# Head injury patterns in helmeted and non-helmeted cyclists admitted to a London Major Trauma Centre with serious head injury

**DOI:** 10.1371/journal.pone.0185367

**Published:** 2017-09-25

**Authors:** Anna E. Forbes, John Schutzer-Weissmann, David A. Menassa, Mark H. Wilson

**Affiliations:** 1 Adult Intensive Care Unit, Department of Critical Care, St Mary’s Hospital, Imperial College Healthcare NHS Trust, London, United Kingdom; 2 Division of Clinical Neurology, Nuffield Department of Clinical Neurosciences, University of Oxford, Oxford, United Kingdom; 3 Imperial Neurotrauma Centre, Imperial College Healthcare Trust Biomedical Research Centre, St Mary’s Hospital, London, United Kingdom; University of New South Wales, AUSTRALIA

## Abstract

**Background:**

Cycle use across London and the UK has increased considerably over the last 10 years. With this there has been an increased interest in cycle safety and injury prevention. Head injuries are an important cause of mortality and morbidity in cyclists. This study aimed to ascertain the frequency of different head injury types in cyclists and whether wearing a bicycle helmet affords protection against specific types of head injury.

**Methods:**

A retrospective observational study of all cyclists older than 16 years admitted to a London Major Trauma Centre between 1^st^ January 2011 and 31^st^ December 2015 was completed. A cohort of patients who had serious head injury was identified (n = 129). Of these, data on helmet use was available for 97. Comparison was made between type of injury frequency in helmeted and non-helmeted cyclists within this group of patients who suffered serious head injury.

**Results:**

Helmet use was shown to be protective against intracranial injury in general (OR 0.2, CI 0.07–0.55, p = 0.002). A protective effect against subdural haematoma was demonstrated (OR 0.14, CI 0.03–0.72, p = 0.02). Wearing a helmet was also protective against skull fractures (OR 0.12, CI 0.04–0.39, p<0.0001) but not any other specific extracranial injuries. This suggests that bicycle helmets are protective against those injuries caused by direct impact to the head. Further research is required to clarify their role against injuries caused by shearing forces.

**Conclusions:**

In a largely urban environment, the use of cycle helmets appears to be protective for certain types of serious intra and extracranial head injuries. This may help to inform future helmet design.

## Introduction

Cycling is an increasingly popular form of transport in London with consecutive year on year increases in the number of cycle journeys made, giving a 61% increase in cycle journeys since 2005 [1]. In 2015, 387 cyclists were killed or seriously injured on London’s roads [2]. Head injury has been shown to be an important cause of mortality and morbidity in cycling accidents [3–5]. It has also been difficult to ascertain exactly which types of head injury cycle helmets are protective for. Few studies provide details of the types of head injuries suffered by cyclists with head abbreviated injury scale (AIS) scores or just presence or absence of injury being more commonly used without specification of injury type [6,7]. Where types of injury are described, differences between helmeted and non-helmeted cyclists are not documented, often due to small numbers [8].

There are no compulsory helmet laws in the UK, although Transport for London (TfL) cycling guidance encourages cyclists to consider wearing a helmet [9]. Recent public health measures for improving cycle safety in London have included improving the infrastructure for cycling and plans to restrict the movement of heavy good vehicles deemed to be dangerous to cyclists, rather than enforcing helmet use [10]. Incidents in which cyclists are injured on London’s roads are decreasing in frequency [2].

The aim of this study was to provide a picture of the spectrum and frequency of specific head injuries suffered by cyclists injured in an urban setting and to identify whether there was a difference in head injury type between cyclists wearing a helmet at the time of their incident and those not. An earlier pilot study, which also analysed cyclists admitted with head injury, suggested that there was a trend towards fewer subdural and extradural haematomas but numbers were insufficient to draw any statistically significant conclusions [11]. A recent paper examining injuries occurring in an urban setting found that helmeted cyclists were less likely to suffer intracranial injury of any type, skull fractures and subdural haematoma but showed no significant difference in the likelihood of sustaining a subarachnoid haemorrhage [12].

## Methods

This was a retrospective observational study. All patients over 16 years old admitted to St Mary’s Hospital London between 1^st^ January 2011 and 31^st^ Dec 2015 for management of serious extra or intracranial head injury sustained whilst cycling (defined as those requiring more than 24 hours admission) were included in the study. Patients admitted for other major trauma who had no head injury, or minor head injury that alone would not have warranted admission for more than 24 hours were not included. This was to ensure that all cyclists included in the analysis had suffered an impact to the head. These patients were identified via the hospital Trauma Registry, which was cross-referenced with Trauma Ward admission lists, Intensive Care Unit admission data and the national Trauma Audit and Research Network (TARN) database. For the period of 1^st^ January 2011- 1^st^ January 2012 the Trauma Registry was not fully established therefore existing data was additionally supplemented with Emergency Department admissions data. Patients who presented more than 24 hours after their injury were not included.

A retrospective analysis of this cohort of head injured cyclists was made. Patients were followed up to the point of hospital discharge.

St Mary’s is a tertiary major trauma centre serving the North West of London. It is one of the four Major Trauma Centres within the major trauma network of London. It became a Major Trauma Centre in December 2010 and data was collected for patients admitted from the start of 2011 once the network was established. It serves a population of approximately three million residents and considerably more who commute in for work. It is the referral centre for six local trauma units and therefore all patients with severe head injury in this region are referred to St Mary’s.

One exception to this group was patients with maxillofacial injury who were referred to Northwick Park Hospital where the specialist beds for isolated maxillofacial trauma are located. Only patients who were transferred as inpatients to Northwick Park were included in the analysis, those discharged home with outpatient follow up were not included.

Data was collected for each head injured cyclist concerning mechanism of injury, type of injury, helmet use, exposure to alcohol and survival to discharge. This was collected from review of Emergency Department documentation, prehospital documentation where available, radiology reports, toxicology results, and patient electronic records.

All radiology reports were viewed initially by a Specialist Registrar and subsequently (or contemporaneously) by a Consultant Radiologist. The Consultant Radiologists report was used to ascertain injury type. Where uncertainty about the presence of a lesion existed within the scan report or clinical correlation was requested, subsequent imaging studies were reviewed alongside documentation from the patient’s admission. Where imaging was not available, patients were not included in analysis.

Data regarding helmet use was collected from pre-hospital documentation and Emergency Department documentation. Where alcohol levels were requested they were recorded as positive or negative. Patients’ exposure to alcohol was not recorded unless a lab value was available. This was to exclude potential bias due to inaccurate reporting of behaviour prior to the accident. Outcome at discharge was recorded as the patient being either alive or dead. This was ascertained from the electronic patient record.

Injuries in head injured cyclists wearing a helmet and those not wearing a helmet were compared. This included both intracranial (defined as contusion (including petechial haemorrhages), subdural haematoma (SDH), extradural haematoma (EDH) and subarachnoid haemorrhage (SAH)) and extra-cranial injury, defined as maxillofacial fractures, skull fractures and lacerations to the scalp or face.

**Fig 1:**
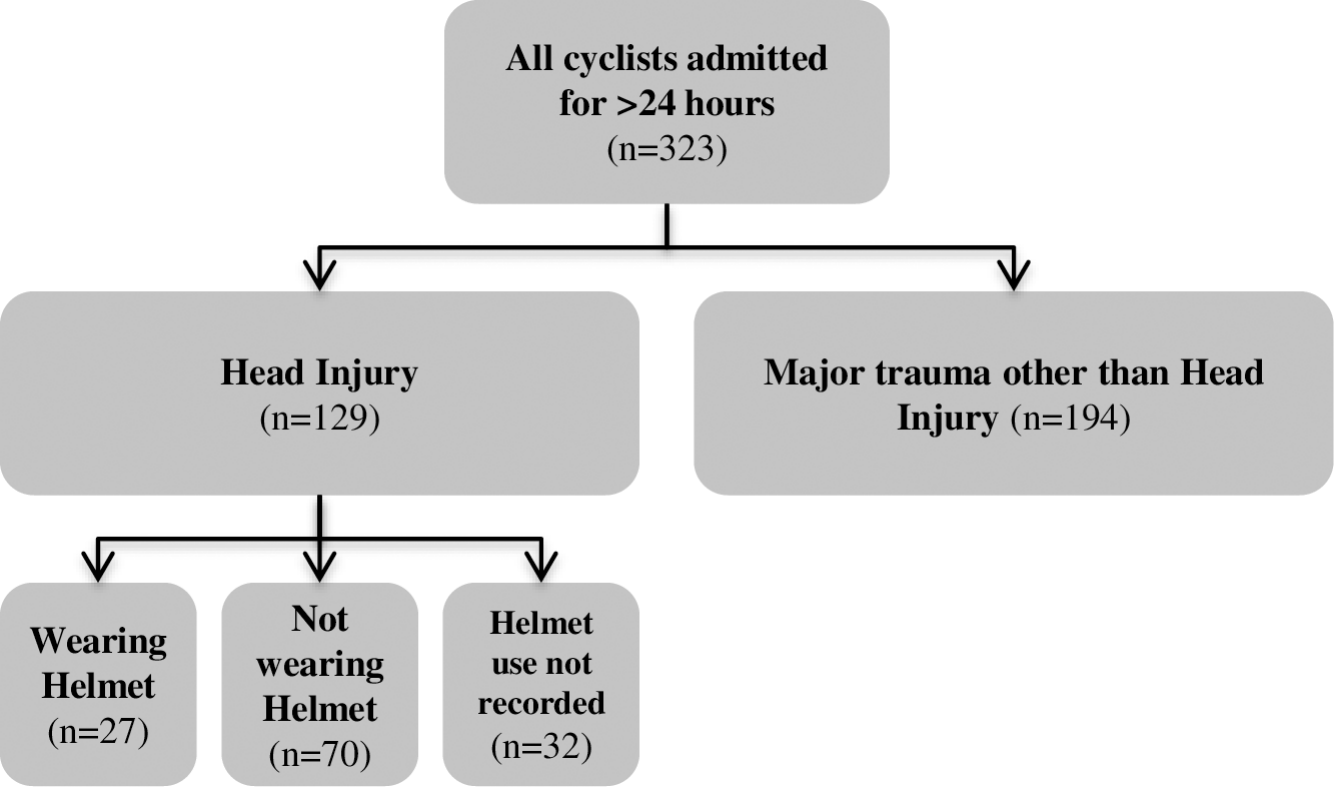
Cyclists included in the analysis.

Comparison of the frequency of each injury type in helmeted and non-helmeted patients was made. Those for whom helmet use was not recorded or unclear were excluded from these analyses (Fig 1).

Glasgow Coma Score (GCS) for each patient was collected from pre-hospital and Emergency Department records. On scene GCS was used in analysis as some patients were intubated by helicopter emergency services on scene so GCS in the Emergency Department was less useful.

### Statistical methods

Data from the helmeted versus the non-helmeted group against each injury outcome were modelled to a logistic regression in Stata® (StataCorp 2017. Stata statistical software Release 15. College station, Texas: StataCorp LLC)) adjusted for age, sex and mechanism of injury. Separate dummies were added for the two older age groups (omitting the youngest as a reference category), male sex, and a dummy for falling (thus using any collision type as the reference category). The adjusted p-values were considered statistically significant for alpha<0.05. Odds ratios and 95% confidence intervals were also calculated using the same software. Mann Whitney U test run in GraphPad Prism (Version 7.0a for Mac OS X, GraphPad Software, La Jolla, California, USA) was used to compare GCS in the helmeted versus the non-helmeted group.

## Results

323 cyclists who were admitted for more than 24 hours were identified. Of these 129 were admitted for management of head injuries under either Neurosurgery, Plastic Surgery, Maxillofacial Surgery or a combination of the three within the Major Trauma Centre. This cohort of head injured cyclists was further analysed. The mean age was 38.9 years (range 16–67), most were in the 25–55 age group and 88% were male. Demographic data, outcome data and mechanism of injury for these patients are displayed in Table 1. In this group the most common injuries were maxillofacial and skull fractures. The least common was extradural haematomas (Table 2). Subarachnoid haemorrhage and contusions were the most common intracranial lesions.

**Table 1:** Demographics of head injured cyclists and mechanism of injury.

		no. of cyclists	%
Age	16–25	19	15
	26–55	95	74
	>55	15	11
Sex	Male	113	88
	Female	16	12
MOI	Car/4x4/van	58	45
	Fall	42	33
	Stationary object	10	8
	Bus	4	3
	pedestrian	3	2.3
	^a^HGV	1	0.7
	Other	11	8
^b^EtOH	positive	20	16
	negative	45	35
	not sent	64	49
Helmet	helmeted	27	21
	non-helmeted	70	54
	not recorded	32	25
Outcome	Dead	4	3
	Alive	125	97

^a^Heavy Goods Vehicle

^b^alcohol level in admission blood sample

**Table 2:** Injury type in cyclists admitted with head injury.

Injury type	no. of cyclists	% of cyclists
Contusions	32	25
^a^SDH	26	20
^a^EDH	13	10
^a^SAH	31	24
Skull fracture	58	45
^a^Max Fax fracture	77	60
Scalp laceration	32	25
Facial laceration	45	35

^a^abbreviations: SDH—subdural haematoma, EDH—extradural haematoma, SAH-subarachnoid haemorrhage, Max Fax–Maxillo facial

Analysis was made of head injured patients who were recorded to have been wearing a helmet (n = 27) and those who were not (n = 70) (Table 3). The remainder had missing or inconclusive recording of cycle helmet use and were therefore excluded from further analysis. The incidence of different injury types in helmeted and non-helmeted cyclists was compared (Table 3). Most patients had more than one injury type. Patients not wearing a helmet were found to be significantly more likely to have suffered intracranial injury (OR 0.2, CI 0.07–0.55, p = 0.002), SDH (OR 0.14, CI 0.03–0.72, p = 0.02) and skull fractures (OR 0.12, CI 0.04–0.39, p<0.0001). No statistically significant differences were identified in any of the other injury types. There was a decrease in the absolute incidence of contusions and SAH in the helmeted compared to the non-helmeted group with a 50% reduction in the odds of contusions and a 74% reduction in the odds of SAH. Although neither p values were <0.05 (contusions (OR 0.5, CI 0.14–1.77, p = 0.28) and SAH (OR 0.26, CI 0.07–1.0, p = 0.05)) in the context of wide confidence intervals, the high p value may be explained by an inadequate sample size. This raises an interesting point about the efficacy of cycle helmets against injuries caused by shearing forces which warrants investigation with a larger study. There was no significant difference in on scene GCS between the groups (p = 0.5). There were four deaths recorded in this study, of these two were wearing a helmet, one was not and one did not have helmet use recorded. Three of them were involved in collisions with motorised vehicles and the mechanism of injury for one was not known. Two were aged 26–55 and two were older than 55 years.

**Table 3:** Injury comparison between helmeted and non-helmeted cyclists with head injury adjusted for age, mechanism of injury and sex.

	Helmet % (n = 27)	No helmet % (n = 70)	OR	CI	p value
Contusions	14.81	24.29	0.50	0.14–1.77	0.28
SDH	7.41	30	0.14	0.03–0.72	0.02
EDH	0	11.43	1.00	1.00–1.00	N/A
SAH	11.11	31.43	0.26	0.07–1.00	0.05
Skull fracture	14.81	57.14	0.12	0.04–0.39	<0.0001
Max Fax fracture	40.74	61.43	0.43	0.17–1.08	0.07
Scalp laceration	18.52	34.29	0.35	0.11–1.12	0.08
Facial laceration	33.33	32.86	1.14	0.42–3.05	0.80
Intracranial injury	25.93	61.43	0.20	0.07–0.55	0.002
Extracranial injury	88.89	95.71	0.33	0.06–1.94	0.22

Comparison of injury types in helmeted and non-helmeted cyclists. Most patients suffered more than one injury type.

## Discussion

This retrospective study of cycling related head injury indicates that helmet use is associated with fewer intracranial injuries, and specifically fewer injuries related to skull fractures.

Limitations of this study were that the exact mechanism of injury, for example direction of impact from vehicle and speed of collision, were not recorded. Additionally, data regarding helmet use was missing in a third of patients. Only patients admitted for over 24 hours were included and hence patients whose helmets may have been “destroyed” with the impact, but who did not sustain significant injury, would not have been represented. Similarly, those who had no need to present to the Emergency Department because of helmet protection and those who died on scene, with or without helmet, are not represented. This study design whereby only cyclists suffering head injury were included in the analysis may introduce bias in underestimating the protective effect of helmets in the absence of a case matched control group with other injuries. For example those who sustained major trauma to other body regions but only minor head injury as a result of the protective effect of helmet use would not be included in analysis.

However, there are some key messages from the results. Injuries associated with direct impact such as subdural haematoma and skull fractures were less frequent in the helmeted group, and none of the helmeted group had an extradural. These are very similar to the results found by Sethi *et al* [12] investigating cycling related head injuries in New York City, both in terms of prevalence of helmet wearing and patterns of injury sustained.

Strengths of this study include the recording of exact injury types. These have not been recorded in other studies where less specific injury severity scores or GCS ranges were used. This more detailed recording of specific injury types may highlight areas of interest for future helmet design.

Our data supports the rejection of the hypothesis that there is no difference between injury types in helmeted and non-helmeted cyclists.

The protection from skull fractures, subdural haematomas and the fact that there were no extradurals in the helmeted cyclists suggests that helmets have greater benefits in protecting from the effects of direct impact rather than the effects of shearing injuries which tend to result in contusions and SAH. It could be hypothesised that this is consistent with what would be expected from the material characteristics of modern helmets. Helmets that absorb energy transfer and minimise the effects of shearing injury, for example, by having sliding outer layers, may afford more protection to these other types of injury. However lower absolute incidence of SAH and contusions in the helmeted group were observed and given the small sample size and large confidence intervals, it is possible that a protective effect exists. Larger studies are required to ascertain this.

Manson *et al* have previously studied London cycling reporting 82% of cyclists activating trauma calls were through cycle versus vehicle collisions [13]. In our series, this accounted for 60% of injuries, and only 3% involved collisions with heavy goods vehicles (HGVs) or buses compared to 26% in the Manson study which analysed all patients presenting as a result of cycle accidents, not just those with head injury. The same group have previously demonstrated that HGVs were associated more with abdominal and pelvic injuries whilst collisions with cars resulted in more head injuries [13].

Our results support the increasing numbers of studies demonstrating that severe brain injury is reduced by helmet use in cyclists. Whilst this may be behaviour related [14], there is increasing recognition it is a real phenomenon thanks to helmet design specifically [3,12].

Early studies collecting data from the 1980’s and 90’s showed a protective effect of helmets against head injury [15–18]. Helmet manufacture, street layout, bicycle and motor vehicle design have all changed a great deal in the last twenty years however more recent studies examining the effect of cycle helmets have continued to show a protective effect [6,7,14].

The majority of these studies used a case control design where controls were those without significant head injury. This could be perceived as a weakness as the mechanism of injury in the control group may not have included any impact to the head. However in our cohort study design patients who may have avoided serious head injury from a head impact by wearing a helmet would be excluded from analysis, potentially introducing bias in under estimating the protective effect of helmets as previously discussed. Despite this a protective effect against some injury types was still demonstrated.

A large meta-analysis examining 40 studies published between 1989 and 2015 which included data from 64, 000 cyclists, showed that helmet use was associated with a 51% decrease in the odds of head injury, a 69% decrease in risk of serious head injury and a 65% decrease in the risk of fatal head injury [19].

Only four deaths were recorded in our series therefore the data set is too small to draw any conclusions about this group, whilst highlighting the low number of deaths after admission to hospital in head injured cyclists which has also been noted in other studies [7,12]. However two of the deaths occurred in people over 55 who represented a very small proportion of the study population. In fact 13% of those older than 55 year died during their hospital admission compared to 2% in the 26–55 year old group and none in the under 25 group. The increased vulnerability of older cyclists has been previously recognised [7, 20].

## Conclusions

This retrospective data analysis of head injured cyclists adds to the existing literature that has demonstrated a lower incidence of certain injuries in cyclists wearing helmets. It demonstrates fewer skull fractures and subdurals in those helmeted cyclists sustaining injury. This study therefore gives weight to the belief that helmets provide protection for some types of head injury, and also highlights the need for research into the protection provided by current helmet design against injuries caused by shearing forces such as contusions and SAH.

## Supporting information

S1 Dataset Raw data for cyclists included in analysis. (XLSX)
